# Recessive congenital myotonia resulting from maternal isodisomy of chromosome 7: a case report

**DOI:** 10.1186/1757-1626-2-7111

**Published:** 2009-04-29

**Authors:** Cristina Bulli, Pier Antonio Battistella, Marta Bordignon, Placido Bramanti, Giuseppe Novelli, Federica Sangiuolo

**Affiliations:** 1 Department of Biopathology, Genetics unit, Tor Vergata University of Rome, via Montpellier, 1, Rome 00133, Italy; 2 Department of Pediatrics, University of Padua, Via Giustiniani 2, Padua 35128, Italy; 3 Department of Clinical Genetics, University of Padua, Via Giustiniani 2, Padua 35128, Italy; 4 IRCCS Centro Neurolesi Bonino-Pulejo, via Palermo SS. 113, Contrada Casazza Messina, Italy

## Abstract

Autosomal dominant (Thomsen) and recessive (Becker) congenital myotonia are two different non dystrophic disorders, due to allelic mutations of the muscle chloride channel gene, located on chromosome 7q35. More than two thirds of the muscle chloride channel gene mutations occur independently in unique families and cause the recessive form of the disease. Becker disease is more common and severe than Thomsen disease. Here, we report on the clinical and molecular data of the first patient with maternal uniparental disomy for chromosome 7 and recessive congenital myotonia. The proband is a 15-year-old male, homozygous for a missense mutation within muscle chloride channel gene, showing few characteristic signs of the Silver Russell Syndrome.

## Introduction

Thomsen (autosomal dominant; OMIM # 160800) and Becker (autosomal recessive; OMIM # 255700) myotonia are characterised by muscle stiffness and myotonia, which is based on an electrical instability of the muscle fiber membrane. The diseases differ clinically by age at onset, by spreading of the symptom myotonia, and by a typical transient muscle weakness only present in the dominant form of the disease. The most prominent symptom of Thomsen's disease is muscle stiffness that starts in early childhood, but it is, however, temporary. The dominant form occurs with a frequency of 1:23,000 to 1:50,000 [1,2]. Both diseases are caused by mutations within the chloride channel gene (CLCN1) gene, coding for the skeletal muscle voltage-dependent Chloride channel and located on 7q35 [3,4]. Up to date 80 different mutations have been reported, the most frequent ones ranging about 0.7% in myotonic patients. Chloride channel mutations are mainly loss-of-function mutations that reduce the stabilizing chloride conductance. Consequently, most of the chloride channel mutations are recessive traits. The few dominant or semidominant mutations act through dominant negative heterodimerization with subunits encoded by the wild-type allele [5,6].

Silver Russell Syndrome (SRS; MIM 180860) is a genetically heterogeneous disease characterised by intrauterine growth retardation, postnatal growth delay, a typical facial appearance with a small triangular face, frontal bossing, thin lips with downturned corners, micrognathia and a variable degree of body asymmetry. Most cases of SRS are sporadic and about 10% of affected individuals shown a maternal disomy for chromosome 7. Cognitive development is variable, with many individuals demonstrating developmental and learning disabilities.

Here we report the first case of Becker recessive myotonia resulting from a maternal isodisomy of the entire chromosome 7.

## Case Presentation

The proband, a Caucasian 15-year-old male, is the only child of healthy, non-consaguineous parents. Family history was unremarkable and pregnancy was uneventful. He was born at the 36^th^ week of pregnancy, with a weight of 2120 g (10^th^-25^th^ centile) and a length at 44 cm (10^th^ centile).

He has never crawled on four limbs but achieved independent walking at the age of 11 months, showing a wide-based, awkward gait with stiffness in lower limbs and difficulty to begin ambulation after a prolonged rest. In the first two years of life, he suffered from two febrile seizure episodes (the EEG findings were normal) and presented language delay (he spoke 10-15 words at 4 years). Since then he started language therapy with appreciable results. A neurocognitive assessment was performed at 4 years and 4 months of age showing a normal range of intelligence (median IQ score on the WPPSI was 95). He attended school with special education needs because of difficulties in reading and writing. At 5 years, extensive investigations were performed: cerebral MRI, echocardiogram, ophthalmological examination and audiometric test were all normal. The electromyography (EMG) revealed the presence of typical myotonic runs (EMG performed in his parents was normal). The serum Creatine Phosphokinase (CPK) was elevated above the normal range (382 U/L, with normal levels being below 270 U/L) in only one occasion, at the age of two years, and reached normal values in the two following measurements. A chest X-ray revealed the presence of dorso-lumbar scoliosis. The thyroid hormonal levels were normal. Muscular biopsy at 5 years revealed mild myopathic change and type I fibers hypotrophy. The FRAXA molecular analysis was negative. He became toiled trained at the expected age. At the age of 7 years he underwent treatment with dyphenilhydantoine for 12 months (the mean dosage was 100 mg/day) but no relief of the myotonia was noticed. Moreover at 10 years a surgical correction for left clubfoot was performed. On careful clinical examination at 15 years, the patient weighted 75.5 Kg (>97^th^ centile), his height was 163 cm (25^th^ centile) and his head circumference was 59 cm (+2.7 SD).

**Figure 1:**
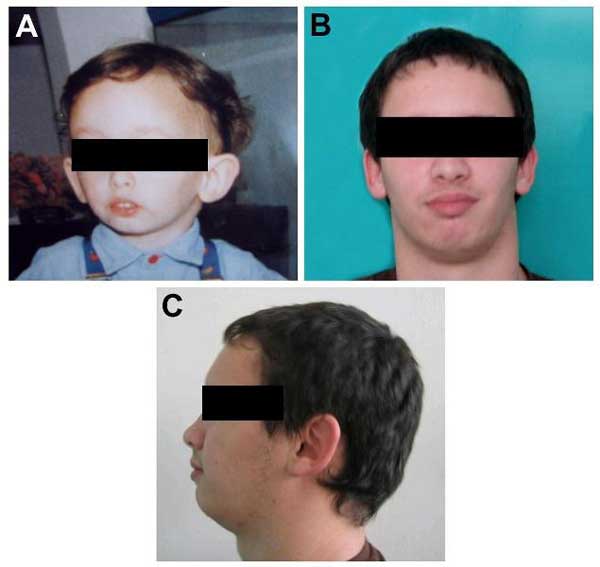
**(A) The patient at the age of 2 years**. The craniofacial dysmorphisms are more relevant at this age. **(B)** and **(C)** The patient at the age of 15 years. Improvement in the facial gestalt is evident.

He presented only few mild cranio-facial dysmorphisms suggestive for SRS: a relative macrocephaly, a small face with a broad forehead, deep set eyes and micrognathia. These clinical findings were more relevant during the first years of life (Figure 1A-C). The palate was high arched and no limb asymmetry was observed. He referred excessive sweating.

During neurological examination, muscle hypertrophy at lower limbs was noticed and myotonia in all four limbs was evident. The boy showed independent walking with lower limb stiffness and weakness; the muscle strength was normal, the Gower's sign was negative; deep tendon reflexes were weak in the upper limbs and brisk in the lower limbs with ankle increased tone.

### Molecular Analysis

**Figure 2:**
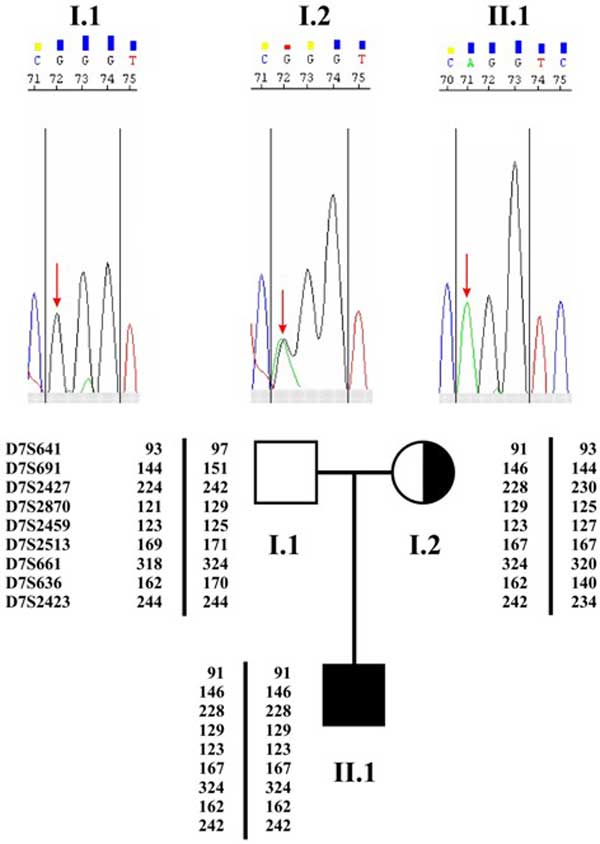
**Family pedigree with the corresponding haplotypes for chromosome 7 and sequence analysis**.

Proband's DNA was screened for CLCN1 mutations together with his non-consanguineous parents, after obtaining signed informed consent. Genomic DNA was extracted from the whole blood of the patient and his parents using the BioRobot EZ1 automated purification (QIAGEN). Sequence analysis of muscle chloride channel (CLCN1) gene on the proband's DNA, showed homozygosity for a G to A transition (c.1063G>A), leading to p.G355R mutation, previously characterised as caused by another nucleotide modification (Figure 2). The mutated allele was segregating only from the mother (heterozygous for the p.G355R mutation) while no mutated allele was detected in the father. Non paternity was immediately excluded using a panel 15 highly polymorphic markers and one marker for amelogenin (AmpFlSTR identifiler). Furthermore, chromosome 7 markers showed an anomalous segregation within the family suggesting an uniparental transmission. A set of nine additional polymorphic markers spanning the entire chromosome 7 (D7S641, D7S691, D7S2427, D7S1870, D7S2459, D7S2513, D7S661, D7S636 and D7S2423) were therefore analysed (Figure 2). Amplifications were analysed on automated sequencer (ABI 3130 Applied Biosystem). PCR were performed in 15 µL reactions containing 100 ng DNA, 1,5 mM MgCl_2_, 150 uM of each dNTP_s_, 150 mM Buffer, 0.15 U Ampli Taq Gold. Thermocycling was performed on a 9700 Applied Biosystems, using the following conditions: an initial denaturation at 95°C for 12 min, followed by 10 cycles each of 94°C for 15 sec, annealing at 55°C for 15 sec and an extension at 72°C for 30 sec, followed by 20 cycles each of 89°C for 15s ec, annealing at 55°C for 15 sec and an extension at 72°C for 30 sec. Finally an extension step at 72°C for 10 min. PCR products were analysed on ABI 3130 XL Gene Mapper (Applied Biosystems). The results unambiguously demonstrated the presence of a maternal isodisomy of the entire chromosome 7 (matUPD7) in the proband (Figure 2).

## Discussion

UPD occurs when an individual inherits two copies of a chromosome pair from one parent, and no copy from the other parent. Our results demonstrate that the proband has two entirely identical chromosomes 7, both inherited from the mother. This could have been due either to a disomic maternal gamete that did not undergo recombination of chromosome 7 during oogenesis or to a post meiotic reduplication or non disjunction of the chromosome 7.

The first observation of UPD in humans was a maternal UPD 7 in two individuals suffering from Cystic Fibrosis (FC), severe growth restriction and growth hormone deficiency, but the face was not typical of SRS [7]. Both patients were isodisomic, and consequently homozygous for a CFTR mutation carried by their mothers.

To date, over 49 cases of maternal UPD7 have been reported. Within these, about 30 characterised by growth retardation have been reported, suggesting that imprinted growth-regulating genes reside on chromosome 7 [8]. In fact maternal UPD7 is predominantly observed among patients with SRS (about 10%) and disrupted genomic imprinting is suggested as the etiological basis for the severe growth retardation [7,8].

On the other hand, only four cases of paternal UPD7 have been reported, all associated with recessive disorders but compatible with normal growth. [9]. All patients had normal pre- and postnatal growth except for one who developed severe postnatal growth retardation, likely due to his serious medical problems. For these reasons, it is possible that most cases of paternal UPD are without phenotypic effects and remain undiagnosed.

In conclusion, chromosome 7 demonstrates imprinting with differing phenotypes for both maternal and paternal UPD. In maternal UPD7, imprinting results in pre and postnatal growth retardation with many of the clinical findings of the SRS. In paternal UPD7, phenotypic effects are limited to autosomal recessive disorders inherited from a heterozygous father, mainly regarding Cystic Fibrosis due to the high CF carrier rate in Caucasian populations.

Our study reports for the first time a patient affected by Becker disease, caused by a maternal isodisomy of chromosome 7. The novel mutation characterised within CLCN1 gene alters an amino acid already reported as modified by another nucleotide substitution, allowing us to consider it a mutation hot spot.

The presence of maternal UPD7 in this patient is causative of both Becker myotonia (due to the two identical copies of the recessive disease-causing gene) and some clinical signs characteristic of SRS (with a craniofacial phenotype being more relevant during childhood) (Fig. 1A-C).

The SRS due to matUPD7 is supposed to be a distinctive subgroup characterized by a milder facial phenotype and additional specific features such as severe speech delay, feeding difficulties excessive sweating and pre-and postnatal growth retardation [10]. The SRS features fade as children grow older.

The patient we have reported about, however, has never presented development delay even though all SRS cases due to maternal UPD 7 reported so far had significant growth retardation.

Although UPD7 appears to be rare, our report suggests that an appropriate genetic counselling at this respect, is indicated within families with individuals heterozygous for CLCN1 mutations.

## List of abbreviations

CLCN1: Chloride Channel Gene; UPD: Uniparental disomy; UPD7: Uniparental disomy for chromosome 7; SRS: Silver Russell Syndrome; EMG: Electromyography; EEG: Electroencephalography; MRI: Magnetic resonance imaging; CPK: Creatine Phosphokinase; matUPD7: maternal UPD7; FC: Cystic Fibrosis; CFTR: Cystic fibrosis transmembrane regulator.

## Consent

Written informed consent was obtained from the patient for publication of this case report and accompanying images. A copy of the written consent is available for review by the Editor-in-Chief of this journal.

## Competing interests

The authors declare that they have no competing interests.

## Authors' contribution

CB conceived of the study and participated in its design and in the interpretation of the data PB, MB, and PB carried out the clinical report. FS and GN reviewed the manuscript critically for important intellectual contents and coordinated the research group. All authors read and approved the final manuscript.
